# Supplementary material for: Detection of a Novel, and Likely Ancestral, Tn916-Like Element from a Human Saliva Metagenomic Library
Source: Genes (Basel). 2020 May 14;11(5):548. doi: 10.3390/genes11050548 (PMC7288454; doi:10.3390/genes11050548)
Supplement: Supplementary file 1 [file genes-11-00548-s001.zip › Figure S1.pdf]

Figure S1: TT31 Sequence

AAGCTTATTATATAACAGTGGAGCGATTACAGAATTAGGAAGCGTGGACAAAGGTACAACGAGGACGGATAA
TACGCTTTTAGAACGTCAGAGAGGAATTACAATTCAGACAGGAATAACCTCTTTTCAGTGGGAAAATACGAAG
GTGAACATCATAGACACGCCAGGACATATGGATTTCTTAGCAGAAGTATATCGTTCATTATCAGTTTTAGATGG
GGCAATTCTACTGATTTCTGCAAAAGATGGCGTACAAGCACAAACTCGTATATTATTTCATGCACTTAGGAAAA
TGGGGATTCCCACAATCTTTTTTATCAATAAGATTGACCAAAATGGAATTGATTTATCAACGGTTTATCAGGAT
ATTAAAGAGAAACTTTCTGCCGAAATTGTAATCAAACAGAAGGTAGAACTGTATCCTAATATGTGTGTGACGA
ACTTTACCGAATCTGAACAATGGGATACGGTAATAGAGGGAAACGATGACCTTTTAGAGAAATATATGTCCG
GTAAATCATTAGAAGCATTGGAACTCGAACAAGAGGAAAGCATAAGATTTCAGAATTGTTCTCTGTTCCCTCTT
TATCATGGAAGTGCAAAAAGTAATATAGGGATTGATAACCTTATAGAAGTTATTACTAATAAATTTTATTCATC
AACACATCGAGGTCAGTCTGAACTTTGCGGAAAAGTTTTCAAAATTGAGTATTCGGAAAAAAGACAGCGTCTT
GCATATATACGTCTTTATAGTGGCGTACTGCATTTGCGAGATTCGGTTAGAATATCGGAAAAGGAAAAAATAA
AAATTACAGAAATGTATACTTCAATAAATGGTGAATTATGTAAAATTGATAAGGCTTATTCCGGGGAAATTGTT
ATTTTGCAGAATGAGTTTTTGAAGTTAAATAGTGTTCTTGGAGATACAAAGCTATTGCCACAGAGAGAGAGAA
TTGAAAATCCCCTCCCTCTGCTGCAAACGACTGTTGAACCGAGCAAACCTCAACAAAGGGAAATGTTACTTGA
TGCACTTTTAGAAATCTCCGACAGTGACCCGCTTCTGCGATATTATGTGGATTCTGCGACACATGAAATCATAC
TTTCTTTCTTAGGGAAAGTACAAATGGAAGTGACTTGTGCTCTGCTGCAAGAAAAGTATCATGTGGAGATAGA
AATAAAAGAGCCTACAGTCATTTATATGGAAAGACCGTTAAAAAAAGCAGAGTATACCATTCACATCGAAGTT
CCACCGAATCCTTTCTGGGCTTCCATTGGTCTATCTGTAGCACCGCTTCCATTAGGGAGCGGAGTACAGTATGA
GAGCTCGGTTTCTCTTGGATACTTAAATCAATCGTTTCAAACTGCAGTTATGGAGGGGATACGCTATGGCTGT
GAACAAGGATTGTATGGTTGGAATGTGACGGACTGTAAAATCTGTTTTAAGTATGGCTTATACTATAGCCCTG
TTAGTACCCCAGCAGATTTTCGGATGCTTGCTCCTATTGTATTGGAACAAGTCTTAAAAAAAGCTGGAACAGA
ATTGTTAGAGCCATATCTTAGTTTTAAAATTTATGCGCCACAGGAATATCTTTCACGAGCATACAACGATGCTC
CTAAATATTGTGCGAACATCGTAGACACTCAATTGAAAAATAATGAGGTCATTCTTAGTGGAGAAATCCCTGC
TCGGTGTATTCAAGAATATCGTAGTGATTTAACTTTCTTTACAAATGGACGTAGTGTTTGTTTAACAGAGTTAA
AAGGGTACCATGTTACTACCGGTGAACCTGTTTGCCAGCCCCGTCGTCCAAATAGTCGGATAGATAAAGTACG
ATATATGTTCAATAAAATAACTTAGTGTATTTTATGATGTTTCTTGCTTCGTTGATACCGCTGGCTAAAGAAATA
CTGAATTTAAAACTTAGTTTATATGTGGTAAAATGTTTTAATCAAGTTTAGGAGGAATTAATTATGAAGTGTAA
TGAATGTAACAGGGTTCAATACAACCAAATTTTAATTTGGCTTTGCATTTTATCTTTTTTTAGCGTATTAAATGA
AATGGTTTTGAACGTCTCATTACCTGATATTGCAAATGATTTTAATAAACCACCTGCGAGTACAAACTGGGTGA
ACACAGCCTTTATGTTAACCTTTTCCATTGGAACAGCTGTATATGGAAAGCTATCTGATCAATTAGGCATCAAA
AGGTTACTCCTATTTGGAATTATAATAAATTGTTTCGGGTCGGTAATTGGGTTTGTTGGCCATTCTTTCTTTTCC
TTACTTATTATGGCTCGTTTTATTCAAGGGGCTGGTGCAGCTGCATTTCCAGCACTCGTAATGGTTGTAGTTGC
GCGCTATATTCCAAAGGAAAATAGGGGTAAAGCATTTGGTCTTATTGGATCGATAGTAGCCATGGGAGAAGG
AGTCGGTCCAGCGATTGGTGGAATGATAGCCCATTATATTCATTGGTCCTATCTTCTACTCATTCCTATGATAAC
AATTATCACTGTTCCGTTTCTTATGAAATTATTAAAGAAAGAAGTAAGGATAAAAGGTCATTTTGATATCAAAG
GAATTATACTAATGTCTGTAGGCATTGAATTTTTTATGTTGTTTACAACATCATATAGCATTTCTTTTCTTATCGT
TAGCGTGCTGTCATTCCTGATATTTGTAAAACATATCAGGAAAGTAACAGATCCTTTTGTTGATCCCGGATTAG
GGAAAAATATACCTTTTATGATTGGAGTTCTTTGTGGGGGAATTATATTTGGAACAGTAGCAGGGTTTGTCTCT
ATGGTTCCTTATATGATGAAAGATGTTCACCAGCTAAGTACTGCCGAAATCGGAAGTGTAATTATTTTCCCTGG
AACAATGAGTGTCATTATTTTCGGCTACATTGGTGGGATACTTGTTGATAGAAGAGGTCCTTTATACGTGTTAA
CCATCGGAGTTACATTTCTTTCTGTTAGCTTTTTAACTGCTTCCTTTCTTTTAGAAACAACATCATGGTTCATGAC
AATTATAATCGTATTTGTTTTAGGTGGGCTTTCGTTCACCAAAACAGTTATATCAACAATTGTTTCAAGTAGCTT
GAAACAGCAGGAAGCTGGTGCTGGAATGAGTTTGCTTAACTTTACCAGCTTTTTATCAGAGGGAACAGGTATT
GCAATTGTAGGTGGTTTATTATCCATACCCTTACTTGATCAAAGGTTGTTACCTATGGAAGTTGATCAGTCAAC
TTATCTGTATAGTAATTTGTTATTACTTTTTTCAGGAATCATTGTCATTAGTTGGCTGGTTACCTTGAATGTATAT
AAACATTCTCAAAGGGATTTCTAAATCGTTAAGGGATCAACTTTGGGAGAGAGTTCAAAATTGATCCTTTTTTT

ATAACAGGAATTCAAATCTTTTTGTTCCATTAAAGGGCGCGATTGCTGAATAAAAGATACGAGAGACCTCTCTT
GTATCTTTTTTATTTTGAGTGGTTTTGTCCGTTACACTAGAAAACCGAAAGACAATAAAAATTTTATTCTTGCTG
AGTCTGGCTTTCGGTAAGCTAGACAAAACGGACAAAATAAAAATTGGCAAGGGTTTAAAGGTGGAGATTTTT
TGAGTGATCTTCTCAAAAAATACTACCTGTCCCTTGCTGATTTTTAAACGAGCACGAGAGCAAAACCCCCCTTT
GCTGAGGTGGCAGAGGGCAGGTTTTTTTGTTTCTTTTTTCTCGTAAAAAAAAGAAAGGTCTTAAAGGTAAGAA
CTTGTATGCTGGGGTGTTGCCCTTTATTTTCAATATTAGTTAAGTACCGTGGGTCAATTTCAATCAATGCTCCCA
CTTGTTCACGAGTTAAACCTCGTTTCAATCGAGCTTCTTTAATGGCTAAACCAAAGGCTCTAAAATCATATTTAT
CTTCTTTTTTACGCATAGTAGACCACCTCTATACATTTTATTGTTCCTACTGAATTAGAAACAGGTATAGAAAAA
CATGTTATATAGTTTATAGGTTCATATTTAATAAAAAGCACTACTAAACGCCAATAAAAAAAACCGTTATATGG
TAGTGCTATTTATGCTGTTAAAATATTGTATCTTACTTCCAAATGGCGGTTTGTTGGAGGTCAAAGTCGCCATG
AAGTATATCACATACAATCAAGTTCCCCACATTGAGTATTTATCAAAAAAAGTCGTCTATCTGCAATAGATAAG
TACGTCCACCAATGTGGTTTTATAAATCATATAGATAGAATAACAGAAGCATGTAAACAGAGAAATAAATCTG
TTTATATGCTTTTTTGGCTATTCAGAACTTTTTTACAAAGTTTATTTATCAGTAATGCAACAAATCCCCCTTTCAC
ATTGGGACTAAGAGTGAAAGGAGATAAACGAGCAAGGCTCACTTCCTTTCCTAGACAGAAAGGGGGTGAGA
AACATGAAACCATCTTCTTTTCAGACCACAATAGAAAATCAGTTTGACTATATCTGTAAACGTGCTATGGAAGA
CGAGCGAAAGAATTATATGCTTTATCTTTCAAGGATTGCAAAGCGTGAGGTGTCCTTTTCGGATGTTGGCGAT
TATCTTGTTAGCCAGTTTGCGACAACAGATAACTATTCAACTGACTTTCAGATTTTTACACTCAATGGGTTATCA
GTAGGCGTTGAAAATGATTTGTTGAGTGAAGCATTACGTGAGTTGCCAGACAAGAAACGTGAAATTCTACTGC
TGTTTTACTTTATGGACATGAGCGATTCAGAAATTGCAGACCTGTTGAAATTGAACCGTTCTACTGTCTATCGG
CATAGAACCAGTGGACTAGCCTTAATTAAAAAGTTTATGGAGGAATTTGAAGAATGAAAACACAATATCCTAT
GATTCCCTTTCCTCTCATTGTAAAGGCAACAGATGGCGATACAGAAGCGATTAACCAGATTCTACATCATTACA
GAGGGTACATAACGAAACGTTCCCTACGACTTATGAAAGATGAATATGGCAATCAAAGTATGGTCGTTGATG
AAGTCTTACGTGGAAGAATGGAAACCAGACTGATTACAAAGATTCTGTCATTTGAAATTAAGTAATAACCTCT
CTCTTTTCGTGGAAGCGTGCCATACTATTCCACGCTTCCCGAACAGGGAGGTTTGTTATTCCATCAAAGCATAT
TGAGCTTTCAATGTGTTTTGATAGGCTAACGAGCCATTGTTCTTTGAAAACTGAATAAAAGTAATTGAATACGT
TTCGATAAGAAAAGAGCCAACGGAGCTAACCGCCATGACCTATCTTCTAAAGATAGCGAGCGTTTCAGTTAGT
GTTCCGAAAAACAATCTTTAGCAGGATTGCCAGCGACGACTTTCTTATCGTGATAATGATACTCCCATACAGTC
AATAGTCCGAGCGTTAAAAGCGTCGCAGGCAATGAGTATGGCTACATGAGAACCATGCAGGGGTGGAACTCC
CGTGAGCTTTGCTAAAGCTGTTCGATTGCTTGTAAAACAACTTTTATGAAATCCAATAAGTGATTTGGAAAGGA
GGATTTTATGAAGCAGACTGACATTCCGATTTGGGAGCGTTATACCCTAACTATTGAAGAAGCGTCAAAATAT
TTTCGTATTGGCGAAAACAAGCTGCGTCGTTTGGCAGAAGAAAATAAAAATGCAAACTGGCTGATTATGAATG
GCAATCGTATTCAGGTTAAACGAAAACAATTTGAAAAAATTATAGATACATTGAACGCAATCTAGCGTAGCCA
AAGGGTCTTGTATATGATAAAATAGTATTAAGTCGTATCAGGGCTCTTTCCATAATGGAAAGGAGCAAATGCC
ATGTCAGAAAAAAGACGTGACAATAAAGGTCGAATTTTAAAGACTGGAGAGAGCCAACGAAAAGACGGAAG
ATACTTATACAAATATACAGATTCATTTGGAGAACCGCAATTTGTTTACTCGTGGAAACTTGTGGCTACAGACA
GAGTACCAGCAGGAAAGCGTGATTGTATCTCACTTAGGGAGAAAATCGCAGAGTTACAGAAAGACATTCATG
ATGGTATTGATGTTGTAGGAAAGAAAATGACACTCTGCCAGCTTTACGCAAAACAGAACGCTCAAAGACCAAA
GGTTAGAAAAAATACTGAAACTGGACGCAAATATCTTATGGATATTTTGAAGAAAGACAAGTTAGGTGCAAG
AAGTATTGATAGTATTAAACCATCAGACGCTAAAGAATGGGCGATTAGAATGAGTGAAAATGGTTATGCCTAT
CAAACCATCAATAACTATAAACGTTCTTTAAAGGCTTCATTCTACATTGCGATACAAGATGATTGTGTTCGGAA
GAATCCATTTGACTTTCAACTGAATGCAGTTCTTGATGATGATACTGTCCCTAAGACCGTACTAACAGGAGAAC
AGGAAGAAAAACTGTTAGCCTTTGCGAAAGCTGATAAAACCTACAGCAAAAATTATGATGAAATTCTGATACT
CTTAAAAACAGGTCTTCGTATTTCAGAGTTTGGTGGTTTGACACTTCCAGATTTAGATTTTGAGAATCGTCTTGT
CAATATAGACCATCAGCTATTGAGAGATACTGAAATTGGGTACTACATTGAAACACCAAAGACCAAAAGTGGT
GAACGTCAAGTTCCTATGGTTGAAGAAGCCTATCAAGCATTTAAGCGAGTGTTAGCGAATCGAAAGAATGAT
AAGCGTGTTGAGATTGATGGATATAGTGATTTCCTCTTTCTTAATAGAAAGAACTATCCAAAAGTGGCGAGTG
ACTATAATGGTATGATGAAAGGTCTTGTTAAGAAATACAACAAGTATAATGATGATAAGTTACCACACATCAC
TCCACATAGTTTGCGACATACATTCTGTACCAACTATGCAAATGCAGGAATGAACCCAAAAGCATTACAATACA

TTATGGGGCATGCAAATATAGCCATGACGCTGAACTATTACGCACACGCAACATTTGATTCCGCAATGTCAGA
GATGAAACGCTTGAATAAAGAGAAGCAACAGGAGCGTCTTGTTGCTTAGTAGTACAAATGAATTTACTACTTA
TTTACCACTTCTGACAGCTAAGACATGAGGAAATATGCAAAGAAACGTGAAGTATCTTCCTACAGTAAAAATA
CTCGAAAGCACATAGAATAAGGCTTTACGAGCATTTAAGAAAATATAAAAAGATAATTAGAAATTTATACTTT
GTTTAATATAAAAAATTACACAATATAATTATCACTTTAAATTTATGTTAACTAACACTATATATAAATTTATTAT
TTCTATAAAAAAAAACTAGAAGCTACTTTATTCACTTCTAGTTTAAATTCTATTTATTTAACATACCTGCTTGAAG
TTGGTACATTTTGTAATAATTACCTTTTAATGCTATCAACTCATCATGTGTTCCAGATTCAATAATTTCACCTTTA
TCTAGTACGTAGATACAGTTAGAATCTTGAATCGTAGATAATCTATGAGCAATCGCTATTGTTGTTCTACCTTTT
CTCATTTTTTCAAGAGAGTTTTGAATCAGCTCTTCTGTTTCAGAGTCGATATTTGCAGTAGCTTCATCAAGAATC
AGGATCTTCGGCTTACTTGCTATAGTTCTAGCAAATGTTAGAAGTTGTCTTTCTCCAGATGAGAATGTTGAACC
TCTTTCTGTAACTACACTATCATACTTATCTTCTAATTTATCTATAAAATTATGTGCATCAACAAACTTCGCCGCT
TCAATAACATCTTCTCTTGTTAGACTTTCATTATACATCTTAATATTAGACTCTACTGTTCCGTGATATAAGAATG
GATCTTGAAGTACTAATCCAACACTGTTTCTTAATTCTTTAGAACTATAATCTTTAATATTAACTCCATCAATTAA
GATTTCTCCTCTATCATAATCATAGAATCTTAAGAATAGATTCATAATAGAAGATTTACCGGAACCAGTAGCTC
CAACAAACGCTATACTCTCTCCATTTTTAACACTAAATGAAATATTTTTAAGAACATCTCGTTTACCATCGTAAG
AGAAGCTAACATTTTTAAACTCGATATCACCACGAGAAATTTTATAATCACTCTCTTTTTGCTCAGGCTCATACT
CTTCATTATCTATTAATGTGAAAACACGACCTGCAGAAATAATAGATGTTTGAAGAATTGAGAAGTTTTGCATA
AGTTCAATTAATGGATTGAACAGCTGATTCGTATATTGAATAAACGCATATATTATCCCAGCAGTAAAACCAGC
ACTTTCAAAAGTTAACCCAAAATACATTACGATAACACCATATGCTAACACTTTAAATAATGCCATAGCTGGTC
TTAATAATATACTATCAACTTTTAATGATTTAGTGTAATAACGAAGGTGTTCATTATTAACCTCTCCAAATTCAT
CAATCAGGCGTTGTTCCTGATTAAATGCTTGGATAATCTTCATACCTTCAATTGATTCAGATAATTTAGTATTCA
TTTCACTAATTTTCGCACGAGAAATTTCTACTAATCTACTAGAAAGCTTCTGATATAAGTATACTGAACCTACCA
TTACAGGAATAAATAGTATCATGTAAATTGTTAATTGCCAACTTAGAGCAATCATCGCAGAAAGTGTTACTACA
AACATAAGTATAGTATTTAAGAAACTTGAGAAAATTGTTCCAAACATATCCGCAACAGCCTGTGTATCATTAGT
TAATCTCGATACGACAGAACCTACAGGAGTTTTATCGAAGTACGACATCCCTAATTTTTGCAAGTTAGCAAAAC
TATCATTACGTAGATCTCTAACTATACTGTGAGCTACTTTTGCGAAGTAATACTCTCCTAAAAATGTAAACACAA
CTCTTAGGATAAACAATCCAAAATAAATTATTAATATATATAATCCAGCTTTAGCAATATTCTTTGTAATATAAT
GATCAATATAATACTGAGCTAAAAGAGGAATTGCTGTTGCGACTATCGAAGTCATTAATATAAATAATAGTGC
TAAAATCGATAATCCTTTATAGCGCAACATATAGCGCATTAATCTAAAAAACGTCTTACTTTTCGACATTTTCAC
TTCCTCCTTTCAAGCTTTCTTCCATCTGTTGACTTTCATAAGTCTCTTTATACCAACCGTTATTGGCGATAAGTTC
ATCATGAGTTCCTTTTTCAATAATTGTGTTATCACCTAAAACAATTATTAAATCAGCTTCCACTACCGCAGAAAG
TCTATGTGCAGTAATGATATTAGTTTGACCACTACGTTGTTCTTTCAAGTTCTCTAAAATAACATGTTCAGTCTT
AGCATCAACCGCAGATAATGAATCATCAAGGATTAAAATTTCAGGATTAATAATTAAAGCACGACTCATCGCT
AAACGTTGTTTTTGACCACCTGAAAGACTAACACCTTTCTCACCAACAATCGTATCAAACTTCTCAGGCATAGC
CATAATATCATCATACAGACCACAAATTTTCGTAATCTCTTCTACCTTCTTATCATCAATTAAAGGATTAGCAAA
ACGAATATTCTCTTTAATCGACATTGCAAATAATACCTGATCTTGAGGTACATATCCGATAAGTTTTCTAAGGTC
ATTAATACTATATTCAGCAATGTCTCTATTGTTAAGCATAATATTACCAGATTTAATATTAAATTCTCTTAATAAC
AGTCTTAATAATGTAGTTTTACCTGCTCCAGTAACACCTACGATACCAAGTGTTTGACCTTTTTCAATAGCAAAC
TTAATATCTCTTAGGACACTCTTACCTTCTTCAAATTCGAAGTTGTTGATATTATACTCAAGTCTACCATTCTCAG
CCTTAACGTTACTATCTAAGTTAGTATTTACCTCATTAACTTCAGCTAATAAACTCTCAATACGTTTATAAGATAC
TTCACCACGTTGTGAAATATTGTATAACCATCCTATTGCTTGAAGCGGCCATACTAACATATCAAGATAAGTCA
CGAAAGTAACTAACTCACCAACAGATAAATCACCATTGGAGATAAAAATACCACCGAAAACAAGAGTAAGTG
TATAAGATAATCCAACGAAGATTAATACCATTGGATCAAATAATGAATTATACTTAGCAGCTATAACATTTTTA
CCAAATACTTTGTTATTAACTTCTCTAAAACTCTTAATCTCATCATTACCATAACCAAATGATTTAGTAACCTTTA
TACCAGAGGCACTTTCTTGAACCTTATTATTCAAGTCAGAAAATGACTCCTGTGCTGCCTCAAAACTCTCATAGT
TTTTATCCCCGATATAATTAGTAGCATACGCTAAAAATGGTAAAGGAATAATTGCTATTAAAGTCAGTTTGAAG
TCAATTAAAAATATCATCGTAAATAATGTCACTAATGCTGTAATACTTGCATCAACCGCTGACATTACTCCACCA
CCAGCAACCATTGCTACAGAATTTATATCATTCGTTGCATGGGCCATTAAATCACCTGTACGGTATTTTTGAAA

GAACGATGGAGACATCTTAGTAAAGTGTTCAAATAATCTTGATCTTAAGATTCTTCCTAAGTTATATGCTGCTC
CAAAAATATAAACACGCCAAACATAACGAAGTGCATACATCGCAAGTGCTGCCAGTACAAGATATCCAACATT
TAAGAATAACTCTCCATTAGTTAAATTTCCAGCCTCAATTTTATCAATAACAGTCCCGATAACTTTCGGTGGAAT
TAAGTTGAAAATACTTACTAAGATAAGGGCAATTATTCCAATAAGATAACGTCTTTTTTCTAGCTTCATAAACC
AAGCTAATTTTCTAAATAAATACATCTACACATCACCTAACCTTTCTCCTAATTTTGAAAATACTTCTCCAATTTT
ACCATTAATTACTAAGTCAGCCATATTGTCCTGAGGAGTAGACGATTTATTAATAACAACTAAATGTTTTCCTCT
AAAGTAATGTAAAAGATTTGCAGCTGGATAAACACTTAATGAAGTACCACCGATAATTAGCACATCAGCTCTT
TCAATAAAGTTAATCGCAGCATTAAATACTGTCATATCTAATTCTTCTTCATATAAAGTAACATATGGTTTTATA
ATTCCACCACATTTATCACAACTCGGTATTTCTTCAGTTTTAGCTAAAAAATCTTCTAAGTTGTAAAAACTTTTAC
ACTTAGTACAATAATTAGCATCAACACTTCCATGAAGTTTCAAAACATTTTTACTGCCTGCTTGTTCATGTAAAG
TATCAATATTTTGTGTAATTACCGCTTTAAGTTTACCTTGTTTTTCTAAATCAGCTAAATAAAAATGAGCTCTATT
CGGTTTAGCATCAGGATATACTAAATGTTTTTTGTAAAAATCAAAGAAATCTACAGGATACTTCACAAACATTG
TTCTAGAAACGAGTTGTTCAGGTGTAAAGTGACGATTTAGTTTAACATTAAACACTCCATTCGCACTTCTAAAG
TCAGGAATATCAGATTCAGTAGAAACACCTGCACCACCAAAAAAGACAATATTATCATTTGATTCTATTATTTC
TCTTAGTTTCTCTATATTATTCATAATACTTACCTCCTTATAAAATATTATAAAAGCCATGAAAAGAAGGAAACC
CTTATATAAGTCTCCTTCTTTCAATTAACTACTAATTAGTTTCTTTATCTTTTTGAGCTTTTAACTCTTTAATACGT
TGCTTACGCTCTTCGATACGAGCTTTTTTCTCTTCACGAGATGCACTCTTAACAGGTCCACGAGCGATATGTTCT
TTACTATCGTTAGTTACAAGGTTAGTTGGTTCTTGTTTCATTTCGATTTCTTCATGACGTTCTACTCTAATACGCA
TTAAGTTAGTAACAACTTCTACCTGAATATCGTCGATCATATCCTCGAACATTTGGTAACCTTCTGTTTTGTATT
CACGAAGTGGATCAATTTGTCCATAAGAACGAAGGAAAATCCCTTTTCTAAGTTGATCCATTTGGTCGATGTG
ATCAGTCCATCTATCATCGATAGCGTTAAGAAGAATGTATTTCTCGAATGAATTCATAGTTTCATCTCCAAGTA
ACTCACGTTTCTCAGCTAACTCAAGATTAATTCTGTGTAATACAAGCTCACGAATTTCATCATCAGACATAACAT
CTGAGTATTCACTTTCAGTAATAGGTTTTTGACCTAAGAACTTCTCATTAAGTGATTTGATGATTTCTTCAGTTT
CTTCTTTTTCACTGTGTGCTTCTAAGTTTTCAGTGATAAATGCCATAGTTTTATCTACTGCTTCACCAATCATCTC
TTGAATAATATCAGTAACTACATCATTTTCTAACACTTCATTTCTTTCAGCGTACATGATTTCACGTTGTTTACGT
AATACATCATCATATTGAAGTACTTGTTTACGTGAGTCATAGTTATTACCTTCAACACGTTTTTGAGCACTTTCA
ACAGATCTACTAACCATTCTACTTTCAAGTGGTGTATCTACATCTTTACCCATGATTTTTTGTAATCTATCAGCTC
CGAAACGAACCATAAGTTCATCTTCTAGAGATAGATAGAATCTACTGTAACCTGGATCCCCTTGACGTCCTGAA
CGACCACGTAATTGGTTGTCAATACGACGAGATTCGTGACGTTCTGTACCGATAACGGCAAGACCACCTAATT
CACGAACACCTTCTCCAAGTTTAATATCCGTACCACGACCAGCCATGTTAGTCGCGATAGTTACTGAACCACGT
TGTCCAGCTTGTTTAATAATTTCTGCTTCACTTTCATTTTGCTTAGCATTAAGTACTTTATGAGGAACACCGTATT
TGTATAATAATTTAGATACTAATTCACTCGTTTCGATAGAAACAGTACCTAAAAGAACTGGTTGTCCTTTATCAT
AACGTTCTTTAACTTCTTGAGCTACTGCATTGAACTTAGCTTCCATATTTGAGTAAATGAAGTCTGGTGCATCAA
TCCTTTGGATTGGTCTGTTCGTAGGAATTGTAGTTACAAACATGTTATAAATGTTTCTAAATTCTTCTTCCTCTGT
TTTACCAGTACCTGTCATACCACTAAGTTTTTTATACATTCTGAAGAAGTTTTGGAATGTAATAGTCGCCATTGT
TTTACTTTCTTTTTGAATCGGCACACCTTCTTTAGCTTCGATAGCTTGGTGTAAACCTTCAGAGAAACGACGACC
AGGCATTGTACGACCTGTAAATTGGTCAACGATTAAAATTTCTCCATCTTCAGCAACTACATAGTCAACATCTA
GTGCCATTGTGTAGTTAGCTTTTAATGCTTGGTTAATGTGGTGAGTTAAATCAACATTTTTTAATTCATAAAGAT
TTTTTAATCCAAAGTAACTTTCCGCTTTATCAATACCATTTTCAGAAAGTTGGATAGCTTTAGTTTTAATATCTAA
AGTATAATCTCCATCACTTCCATCTTCTTCCTCTGCTTTTTTCAGAGTTTTAACGAAGGCATTTGCAACTTGGTAT
AATGAAGTTGATTCTTGACCTTCACCAGAGATAATAAGTGGTGTACGTGCTTCATCGATAAGCACTGAGTCAA
CCTCATCGATCACAGCATAGTTTAATGGACGTTGAACACGCGCTTCAACTGTTTTAACCATGTTATCTCTTAAGT
AGTCAAACCCTAACTCGTTATTTGTTGAGTATGTAATATCAGCGTTATATGCTGCTCTTTTCTCTTCAGAGTTTA
AAGAGTTTAAGTTAAGACCTACAGAAAGTCCCATGTAGTTATAGAAAACTCCCATCTCTTGAGCGTCACGTTG
AGATAAGTATTCGTTAACTGTAACAACGTGTACACCTTCACCTGCTAATGCGTTTAAGTATACTGGCATTGTCG
CAGTAAGAGTTTTACCTTCCCCTGTTCTCATCTCCGCGATATCCCCACGGTGAAGTGCGATACCACCCATGATTT
GAACTTTATATGGTTTCATTCCTAGAGAACGTAGTGCACCCTCACGAGCTGCAGCAAACGCATCTACTAAAATT

TTATCTAATATATCCGGTGTATCTTTACCTTTTTCTTTTTGTTCTTGAATATAAGCTTTAAATTCTTCTGTTTTATTT
ACTAATTCTTCATCGCTAAGATTTGAATATTCTTCATCTTTAGCTAATACTTTATCAGCTAATTTTGATAAGCTT
