# Supplementary material for: Detection of a Novel, and Likely Ancestral, Tn916-Like Element from a Human Saliva Metagenomic Library
Source: Genes (Basel). 2020 May 14;11(5):548. doi: 10.3390/genes11050548 (PMC7288454; doi:10.3390/genes11050548)
Supplement: Supplementary file 1 [file genes-11-00548-s001.zip › Table S1.pdf]

Table S1: Primers used in this study.

Name	Sequence (5’-3’)	Purpose	Source/Reference
pCC1-F	GGATGTGCTGCAAGGCGATTAAGTTGG	End sequencing of pCC1BAC	Epicentre®
pCC1-R	CTCGTATGTTGTGTGGAATTGTGAGC	End Sequencing of pCC1BAC	Epicentre®
TT31_F1	GGTTGACTCAGTGCTTATCG	Sequencing of TT31	This Study
TT31_R1	GAGGTCAGTCTGAACTTTGCG	Sequencing of TT31	This Study
TT31_F2	GAAGATGGAAGTGATGGAG	Sequencing of TT31	This Study
TT31_R2	GCAAACGACTGTTGAACC	Sequencing of TT31	This Study
TT31_F3	CTATCGCGACTAACATGG	Sequencing of TT31	This Study
TT31_R3	CTGCTCGGTGTATTCAAG	Sequencing of TT31	This Study
TT31_F4	CGTAATGGTTGTAGTTGC	Sequencing of TT31	This Study
TT31_R4	CGATTGGTGGAATGATAGC	Sequencing of TT31	This Study
TT31_F5	GCTAAACCGAATAGAGCTC	Sequencing of TT31	This Study
TT31_R5	GCTTTCGTTCACCAAAACAG	Sequencing of TT31	This Study
TT31_F6	CGAAAGTTATCGGGACTG	Sequencing of TT31	This Study
TT31_R6	CTTTGCTGAGGTGGCAG	Sequencing of TT31	This Study
TT31_F7	GTTAGTATGGCCGCTTCAAG	Sequencing of TT31	This Study
TT31_R7	CCATCTTCTTTTCAGACC	Sequencing of TT31	This Study
TT31_F8	CGTTTAGCGATGAGTCGTG	Sequencing of TT31	This Study
TT31_R8	CTAACGAGCCATTGTTC	Sequencing of TT31	This Study
TT31_F9	GTAGGTTCTGTCGTATCG	Sequencing of TT31	This Study
TT31_R9	GTCGTATCAGGGCTCTTTCC	Sequencing of TT31	This Study
TT31_F10	CTTACGATGGTAAACGAG	Sequencing of TT31	This Study
TT31_R10	CCGTACTAACAGGAGAACAG	Sequencing of TT31	This Study
TT31_R11	CGCTGAACTATTACGCACAC	Sequencing of TT31	This Study
